# A Full Mouth Rehabilitation of an Asthma-Associated Patient With Tooth Surface Loss: A Case Report

**DOI:** 10.7759/cureus.61051

**Published:** 2024-05-25

**Authors:** Khadijah M Baik

**Affiliations:** 1 Department of Prosthodontics, Faculty of Dentistry, King Abdulaziz University, Jeddah, SAU

**Keywords:** occlusal vertical dimension, extracoronal restoration, prosthodontics, asthma, double veneers, full mouth rehabilitation, tooth surface loss

## Abstract

Tooth surface loss (TSL) is multifactorial and, when severe, it can severely impact the quality of life. Although carbonated soft drinks, with their high sugar and acid content, are a common cause of TSL, the effects do not usually mandate full-mouth rehabilitation. Nevertheless, when combined with other factors such as parafunctional habits or other drivers of high consumption, TSL can be severe. Here we present the case of a 35-year-old man who presented with mild to severe TSL throughout the oral cavity caused by erosion and attrition. Causative factors were heavy consumption of soft drinks for years to quench thirst caused by asthma, side effects of asthma-related medications, and other parafunctional habits. The eight-step approach was as minimally invasive as possible, considering the severe presentation of TSL, and offered the patient a more durable treatment option than previously provided resin-based composite restorations. Presenting this case allows us to discuss the causes of TSL and also describe full mouth rehabilitation of TSL at increased occlusal vertical dimension with indirect restorations. We also demonstrate the integration of removable and fixed options, when progressing complex restorative cases.

## Introduction

Tooth surface loss (TSL) is a normal physiological process that happens throughout life, but it is considered pathological when it is rapid [1]. TSL occurs due to risk factors that cause erosion, attrition, abrasion, abfraction, or their combination [2], and it can either be localized or generalized. If TSL progresses slowly, then teeth undergoing surface loss (or the opposing teeth) can supererupt, reducing the interocclusal space for subsequent restorations.

However, if TSL progression is rapid, which is rare, then the interocclusal space may be maintained, and treatment may not require compensation for the missing interocclusal space. Establishing the cause and controlling progression are mandatory first steps when treating TSL. In most cases, to compensate for the lack of interocclusal space, a space should be created by orthodontic intrusion, crown lengthening, and intentional root canal treatment (to allow for additional tooth reduction) or by increasing the occlusal vertical dimension (OVD) with a bite splint. Dahl et al. [3] first introduced this method, reporting the use of a removable cobalt-chromium anterior bite splint in a patient with localized attrition, to provide space for subsequent restoration. Using a bite splint in the anterior area provides similar orthodontic intrusion anteriorly and orthodontic extrusion posteriorly.

Here, we present a case of mild to severe TSL (according to Smith and Knight criteria [2]) in a patient with asthma, treated with extensive extracoronal restorations at increased OVD to re-establish esthetics and function. Presenting this case allows us to discuss the causes of TSL, describe full mouth rehabilitation of TSL at increased OVD with indirect restorations, and illustrate how removable and fixed options can be integrated when progressing complex restorative cases.

## Case presentation

**Figure 1:**
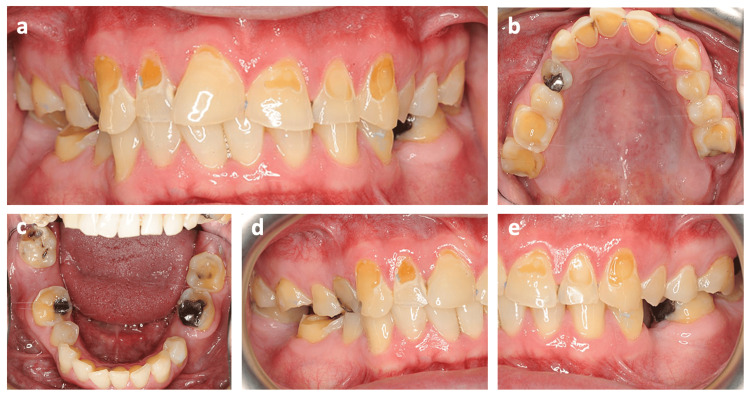
Intra-oral photographs of the patient on the first presentation to the clinic. (a) Frontal view, (b) upper occlusal view, (c) lower occlusal view, (d) lateral view (right), (e) lateral view (left).

A 35-year-old White man presented to the clinic unable to smile due to unsightly teeth and concern about losing his teeth (Figure 1). Although the problem had started eight years previously, over the last three years, he had developed sensitivity to cold of the upper anterior teeth. Two years ago, his general dental practitioner had attempted to cover his sensitive teeth with direct resin-based composite restorations, but these repeatedly failed despite multiple applications.

Medically, he had a history of asthma since the age of 18, for which he was taking corticosteroids and salbutamol through inhalers. Apart from asthma, he was otherwise healthy. Since his diagnosis, he had experienced excessive thirst, for which his doctor advised drinking plenty of fluids. However, he had overcome his thirst by choosing to drink three liters of sweet, carbonated soft drinks daily for the past 10 years. His uric acid levels were also raised.

Dentally, he had been diagnosed with generalized marginal gingivitis and multiple carious (UR4, LR8) and missing (LL5, LR7) teeth. He also had generalized severe TSL of the maxillary anterior teeth, molars, and mandibular molars, as well as localized mild TSL of the maxillary premolars caused by erosion and attrition. TSL severity was graded according to the four-point Smith and Knight tooth wear index [2], where zero indicates no tooth wear and four indicates severe tooth wear. All teeth tested vital, and there was group function occlusion on both the right and left sides.

Primary impressions and a facebow record were obtained to mount the study models on a Denar Mark II semi-adjustable articulator (Whip Mix Corporation, Louisville, USA). Using occlusal bite registration on the retruded contact position (RCP), the mandibular cast was mounted in relation to the maxillary cast. Diagnostic wax-up of maxillary and mandibular teeth at 3 mm increased OVD was performed to compensate for the lack of interocclusal space due to TSL.

The patient’s prosthodontic treatment plan (see summary in Table 1) aimed to provide indirect restorations at 3 mm increased OVD, including double veneers (metal palatal and ceramic labial) from upper right to upper left canines, E-max crowns (Ivoclar Vivadent Inc., Schaan, Liechtenstein) for the upper right and left premolars, and gold onlays for UR6, UR7, UL6, LR6, LL6, and LL7. Maintenance treatment included an occlusal stabilizing splint and review on a regular basis. To prevent TSL progression, the patient was advised to cut down on sugary and acidic drinks, dilute them with water, or use a straw. To control caries progression, he was prescribed 5000 ppm fluoride toothpaste.

**Table 1:** Overview of the treatment plan. TSL: tooth surface loss; OVD: occlusal vertical dimension

Stage	Plan
1	Prevent the progression of TSL, control caries progression, and restore carious teeth
2	Increase the OVD by 3 mm using a removable splint
3	Test the increased OVD after one to two months
4	Restore the teeth in a posterior to anterior direction, first by fabricating gold onlays for posterior teeth, while still wearing the splint from premolar to premolar
5	Fabricate ceramic crowns for upper premolars at the established increased OVD, while still preserving anterior space by using an occlusal splint
6	Minimally prepare the palatal parts of the upper anterior teeth for indirect gold veneers, thus restoring all teeth with indirect restorations at an increased OVD of 3 mm, then discarding the splint
7	Restore the labial surfaces of anterior teeth with ceramic veneers
8	Protect the costly lengthy comprehensive treatment with night guard from parafunctional habits

To increase the OVD, the patient was provided with a maxillary occlusal Michigan splint at 3 mm increased vertical dimension. The patient was followed up six weeks later, and he had adapted well to the increased OVD, suffering only minor discomfort over the first week.

**Figure 2:**
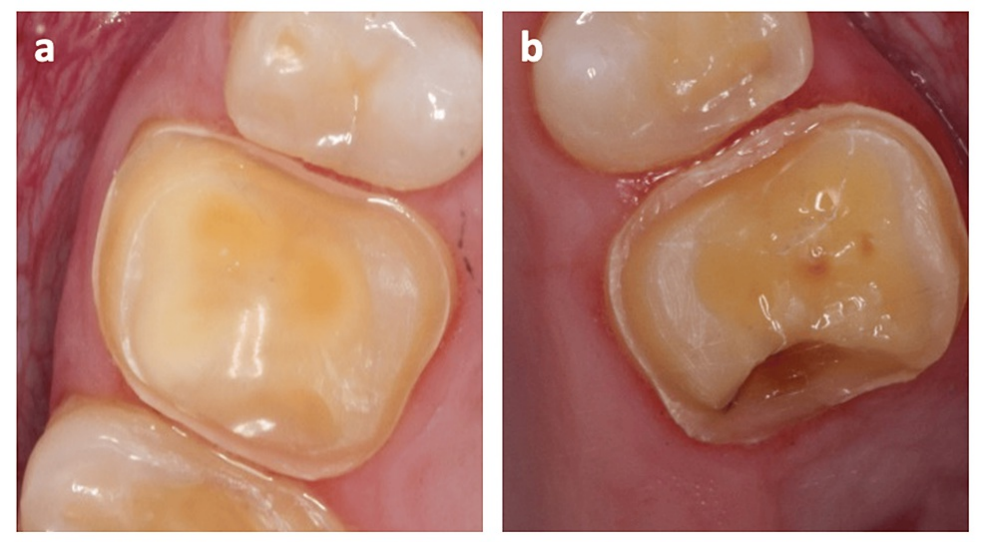
Onlay preparation for UR6 (a) and UL6 (b).

**Figure 3:**
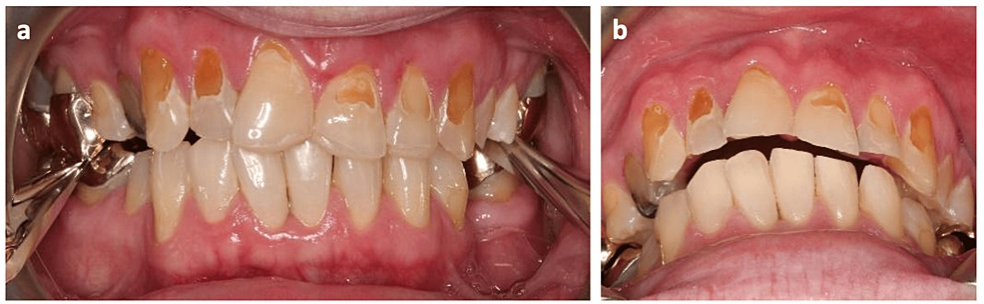
Verification of occlusal contact posteriorly (a) and 3 mm space established anteriorly (b).

Following caries excavation and composite restorations, gold onlays were prepared for UR6, UR7, and UL6 (Figure 2). A definitive impression was made in a special tray with additional silicone medium and a light body. Prepared teeth were temporized, and the laboratory was instructed to fabricate gold onlays, which were subsequently tried for fit, marginal adaptation, occlusion, and intercuspal contacts before cementation with Rely-X Unicem (3M, St. Paul, USA). Gold onlays were then completed for LR6, LL6, and LL7, another impression was made, and preparations were temporized for the right side. The occlusal splint was cut back in the molar area so that the patient could still wear it to stabilize the established premolar to premolar OVD, and the final onlays were cemented on the molars at the increased OVD. Occlusion was checked, ensuring even occlusal contacts in the molar area and maintenance of the occlusal space anteriorly by the occlusal splint (Figure 3).

UR4, UR5, UL4, and UL5 were prepared for E-max lithium disilicate crowns, and provisionals were cemented. Crowns were tried as previously and cemented using Rely-X Unicem.

Finish lines were defined for the indirect metal veneers. Exposed sclerotic dentine was covered with dentine bonding agents. Impressions were made, and preparations were temporized. At this point, the patient was instructed not to wear the occlusal splint, as occlusion was established for the entire arch. 

**Figure 4:**
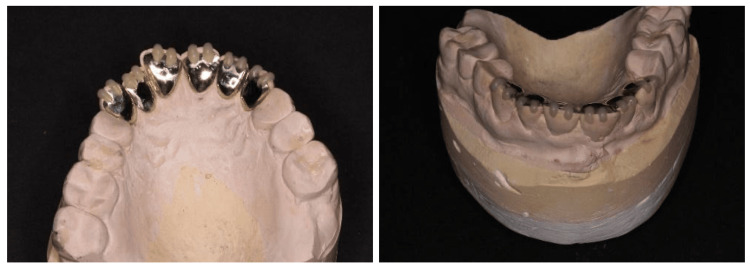
Gold palatal veneers with composite incisal hooks.

Gold palatal veneers were checked for marginal accuracy, occlusion, and interproximal contacts, retaining a 1 mm space incisally for the labial overlap of labial ceramic veneers. Veneers were adapted and fitted with the help of laboratory-fabricated composite incisal hooks, which were removed after cementation (Figure 4).

**Figure 5:**
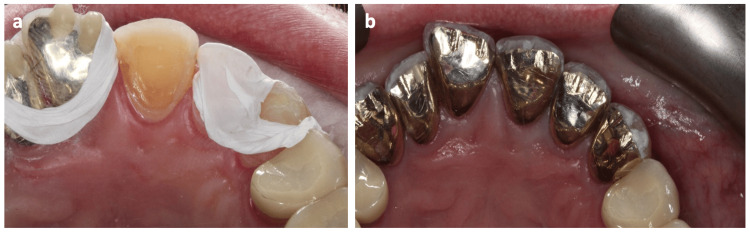
(a) Separation using PTFE tape. (b) Cemented gold palatal veneers. PTFE: polytetrafluoroethylene

The internal surface was treated with 50-micron aluminum oxide sandblasting after try-in and prior to cementation. Teeth were etched using phosphoric acid, bonded using a dentine bonding agent, and cemented with Panavia 21 resin cement (Kuraray America Inc., Houston, USA). A polytetrafluoroethylene (PTFE) tape was used for separation (Figure 5).

Prior to labial veneer preparation, the worn labial surfaces of UR3, UR2, UR1, UL1, UL2, and UL3 were restored with resin-based composite to facilitate veneer preparations. To enhance the marginal level for UL1, electrocautery was performed prior to veneer preparation. Electrocautery was performed using electrosurgical electrodes and was preferred over using a scalpel to minimize bleeding (due to the ability of electrocautery to cut and coagulate soft tissues at the same time) so that veneers could be prepared at the same visit. Labial veneer preparation was completed from canine to canine, followed by impressions and provisional restorations. The laboratory was instructed to fabricate feldspathic ceramic veneers from canine to canine. Veneers were tried using Calibra Light try-in paste (Dentsply Sirona, Charlotte, USA). The fitting sequence was also recorded for definitive cementation. The patient confirmed that he was satisfied with the esthetics. 

The fitting surfaces of the veneers were etched with 4% hydrofluoric acid (BISCO Inc., Schaumburg, USA) for four minutes, washed, and a silane coupling agent was applied and allowed to dry. Meanwhile, teeth were etched with 35% phosphoric acid (Ultradent Products Inc., South Jordan, USA) and bonded with the dentine bonding agent. Calibra Resin Cement was used for final cementation. Occlusion and lateral excursive and protrusive movements were checked.

**Figure 6:**
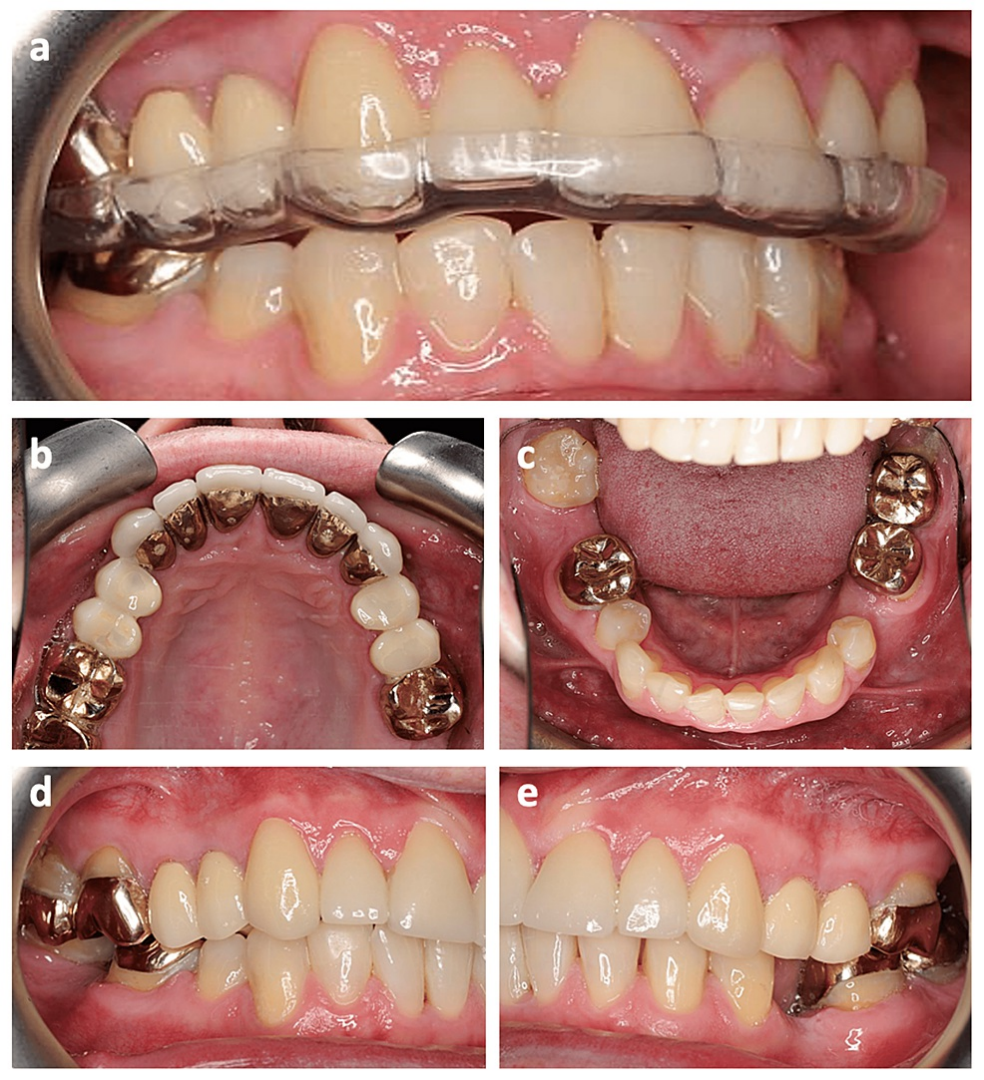
The completed case. (a) Stabilizing splint, (b) upper occlusal view, (c) lower occlusal view, (d) lateral view (right), (e) lateral view (left).

Maxillary and mandibular alginate impressions, a facebow record, and occlusal bite registration were taken for occlusal stabilizing splint fabrication. The lab was instructed to mount the models on a semi-adjustable articulator and provide a splint with multiple contacts all around the arch and canine risers. The splint was tried in, and occlusion was checked and adjusted to complete the management (Figure 6).

## Discussion

Here we restored multifactorial TSL, previously treated non-invasively, with full mouth rehabilitation and restoration of esthetics, function, and occlusion. In this case, the patient had multiple carious and missing teeth, generalized mild to severe TSL, and loss of the vertical dimension that had severely affected his social and functional quality of life. The treatment plan involved controlling both caries and TSL, restoring carious teeth, increasing the OVD by 3 mm as determined by the diagnostic wax-up, and restoring the teeth by indirect restorations at the increased OVD.

Following a thorough diet analysis, the cause of TSL was determined to be erosion and attrition. Erosion was caused by two factors: heavy consumption of sweet, acidic carbonated drinks [4] to overcome thirst caused by asthma, together with his asthma medications. Asthmatic patients tend to feel excessively thirsty [5], and about 60% of them try to overcome this by drinking soft drinks. Additionally, asthma medications can have a significant impact on erosive TSL [6-8], thought to be due to the low pH of the inhalers (pH 5.5) which, if used several times a day, can cause erosive TSL that physiologically begins at pH 5.5 [9]. Moreover, many asthma patients suffer from gastroesophageal reflux [10], with a prevalence as high as 80% (many cases of which are clinically silent) [11], which further exacerbates the acidic intraoral environment. Our patient was advised to cut down on sugary carbonated drinks [12], dilute them with water, consume them once daily rather than throughout the day, and wait one to two hours before brushing after consuming soft drinks, as previously recommended [13].

The management plan was decided after the exhaustion of non-invasive treatment options. While direct composite restorations can offer a high survival rate [14], as this patient had already received multiple composites, it was decided that indirect restorations would be a more reliable treatment option at an increased OVD of 3 mm. 

Although some patients may not accept the increase in vertical dimension, it is usually successful if performed gradually or is less than 5 mm. Signs and symptoms developing during the process are usually temporary and resolved [15]. In this case, the patient only experienced discomfort over the first week. 

After establishing the increased OVD, the approach was to restore the posterior teeth first, as loss of posterior tooth support is often the main cause of OVD loss [16]. The aim was to maintain the occlusion throughout the restoration of the posterior teeth, followed by esthetic restoration of the anterior teeth. We used a fixed-removable combination to progress the treatment. While restoring the upper and lower posterior teeth, the patient still wore the splint to maintain the space anteriorly. The patient stopped wearing the occlusal splint when the canine-to-canine palatal surfaces were provisionally restored. 

As gold indirect restorations have a reported 100% survival at 50 years [17], gold onlays were selected to restore the molars, especially considering the attrition factor of the TSL. The decision to use the sandwich technique (double veneers) anteriorly was driven by a desire to conserve as much tooth structure as possible and to preserve interproximal tooth structure that would have been lost by using a full crown. Additionally, only a slight definition of the finish line was performed on the palatal surface. In contrast, on the labial surface, erosive lesions were restored first with resin-based composites, after which a veneer preparation was performed. 

After tooth preparation, a dentine bonding agent was applied to improve the bonding strength of the final restoration [18] and to reduce postoperative sensitivity [19], although a recent systematic review reported no significant difference between immediate and delayed dentine sealing [20]. Finally, the patient was provided with an occlusal splint to protect the indirect restorations from chipping associated with parafunctional habits.

This case showcases a comprehensive full mouth rehabilitation with indirect restorations. While it may seem invasive, tooth preparations were minimal, as the teeth were already eroded by TSL. The choice of gold is by itself very conservative when compared with ceramics. Nevertheless, such extensive treatment is costly and might not be accessible to all. A cheaper treatment option might have been possible if the patient’s physician had clarified that it is important to avoid sugary drinks to quench thirst, the patient sought professional dental care earlier, and if the progression of TSL had been prevented at an earlier stage. In that case, the patient may have been managed with only simple direct composite restorations.

## Conclusions

This case illustrates the importance of early prevention and transition from a non-invasive to an invasive treatment plan in a timely manner and after exhausting all non-invasive options. It also highlights the importance of thorough planning of full mouth rehabilitation cases and testing each step of treatment. Our treatment offered the patient a smooth transition by first increasing the vertical dimension using a removable splint over a period of weeks followed by fixed final restorations. This approach achieved excellent esthetic and functional outcomes, and the patient was satisfied.

This case also highlights a few important points when treating full mouth rehabilitation cases of TSL: first, to establish and control the disease progression; second, to exhaust all non-invasive treatment options before proceeding to indirect restorations; third, to still select whenever possible the least invasive indirect restoration, especially in TSL cases where tooth structure has already been lost due to the process; fourth, to take enough time to plan the case and allow the patient to cope with the treatment; fifth, to select the best option for restoring posterior or anterior teeth first, according to the case; and finally, to protect the restorations and follow up the patient regularly.
